# Supplementary material for: Interface engineering of charge-transfer excitons in 2D lateral heterostructures
Source: Nat Commun. 2023 Apr 28;14:2438. doi: 10.1038/s41467-023-37889-9 (PMC10147613; doi:10.1038/s41467-023-37889-9)
Supplement: Supplementary file 1 — Supplementary Information [file 41467_2023_37889_MOESM1_ESM.pdf]

Supplementary Information for
Interface engineering of charge-transfer excitons in 2D lateral heterostructures

Roberto Rosati,[1] Ioannis Paradisanos,[2] Libai Huang,[3] Ziyang Gan,[4,5] Antony George,[4,5] Kenji Watanabe,[6] Takashi Taniguchi,[7] Laurent Lombez,[2] Pierre Renucci,[2] Andrey Turchanin,[4,5] Bernhard Urbaszek,[2,8] and Ermin Malic[1]

[1]*Department of Physics, Philipps-Universität Marburg, Renthof 7, D-35032 Marburg, Germany*
[2]*Université de Toulouse, INSA-CNRS-UPS, LPCNO, 135 Avenue Rangueil, 31077 Toulouse, France*
[3]*Department of Chemistry, Purdue University, West Lafayette, IN, USA*
[4]*Friedrich Schiller University Jena, Institute of Physical Chemistry, 07743 Jena, Germany*
[5]*Abbe Centre of Photonics, 07745 Jena, Germany*
[6]*Research Center for Functional Materials, National Institute for Materials Science, 1-1 Namiki, Tsukuba 305-0044, Japan*
[7]*International Center for Materials Nanoarchitectonics, National Institute for Materials Science, 1-1 Namiki, Tsukuba 305-0044, Japan*
[8]*Institute of Condensed Matter Physics, Technische Universität Darmstadt, 64289 Darmstadt*

1. ENVIRONMENT-DEPENDENT CHARGE TRANSFER EXCITONS

In the main manuscript, we have shown how charge transfer (CT) excitons are crucially affected by the dielectric environment. Here, we further analyze this dielectric-screening-dependent behaviour. Figure S1(a) shows the photoluminescence (PL) at 30 K for the hBN-encapsulated ($\varepsilon = 4.5$) and free-standing ($\varepsilon = 1$) case, cf. the blue and red line, respectively. While the hBN-encapsulated lateral heterostructure shows a clear low-energy feature X_{CT} stemming from CT excitons (blue), only the monolayer peak X_{Mo} is visible in the free-standing case (red). Crucial for the difference between free-standing and hBN-encapsulated results is the different energy separation $X_{Mo} - X_{CT}$ between CT and monolayer excitons, which drastically increases for a larger dielectric screening (cf. Fig. S1(b) and Fig. 3(a) in the main manuscript).

Besides exciton optics, the hBN-encapsulation of lateral heterostructures could lead to a number of possible technological applications in optoelectronics. The large energy separation between the monolayer and charge transfer excitons ($X_{Mo} - X_{CT}$), Fig. S1(b) results in a one-dimensional excitonic confinement, which could trap excitons and trigger an efficient dipole-driven exciton transport [1]. Furthermore, optoelectronic devices rely on an efficient charge separation. This becomes realistic in hBN-encapsulated lateral heterostructures due to the small binding energy of CT excitons (X^b_{CT}, blue line of Fig. S1(c) and Fig. 3(b) in the main manuscript). Importantly, our work predicts for the hBN-encapsulated case a large $X_{Mo} - X_{CT}$ and a small X^b_{CT} for typical band-offsets of approximately 200 meV [2], cf. Fig. S1(b-c). The CT binding energy is about one order of magnitude larger for the free-standing case than for hBN-encapsulation, cf. Fig. S1(c). This can be explained by a more efficient screening of the Coulomb interaction in hBN-encapsulated samples, resulting in a weaker attraction of Coulomb-bound electrons and hole. As a direct consequence, CT excitons in hBN-encapsulated samples have a one-order-of-magnitude larger dipole moment compared to the free-standing case, cf. Fig. S1(d).

The large dipoles induce the decrease of binding energies. In Fig. S1(d), we investigate the relation between the dipole and the binding energy by exploiting its dependence on the dielectric constant from Fig. 3 in the main manuscript. For increasing dipoles $d_{e\text{-}h}$, we observe a monotonic decrease of the binding energy - qualitatively similar to the one reported in Ref. [3] for suspended vertical MoS_2-WSe_2 heterostructure with increasing interlayer separation. This indicates that an increased electron-hole separation reduces the binding energy. CT excitons, however, show a much larger dipole than interlayer excitons, because for the latter the separation is limited to the interlayer distance. As a result, the large dipole of CT excitons for hBN-encapsulated lateral heterostructures is responsible for their

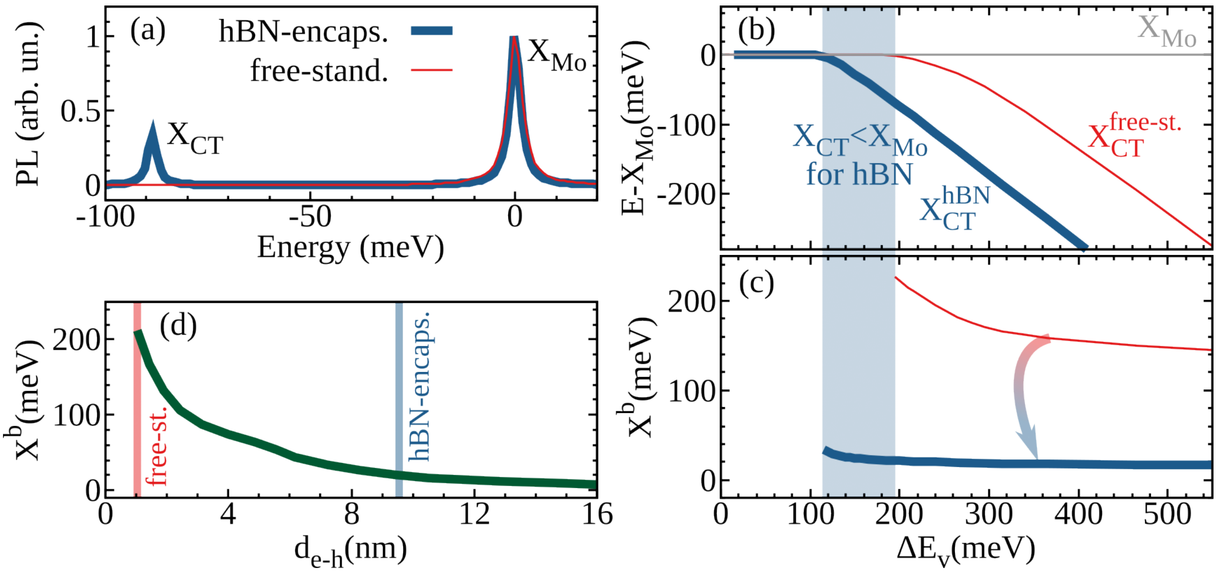

Fig. S1: **Environment-dependent charge transfer (CT) exciton PL.** (a) Photoluminescence, (b) energy of the lowest exciton E and (c) CT exciton binding energies X^b comparing hBN-encapsulated (blue) and free-standing lateral heterostructures (red). In (b,c) we consider different band offsets ΔE_v, with the blue area indicating when CT excitons are the lowest-energy state under hBN-encapsulation but not for the free-standing case. Under hBN-encapsulation the CT-exciton binding energies are strongly reduced compared to the free-standing case. This is due to the large in-plane dipoles of CT excitons and because the binding energies decrease with increasing dipole, as shown in (d) for the case w =2.4 nm and ΔE_v=215 meV.

strongly reduced binding energy of about 20 meV. Considering substrates with reduced dielectric screening, the stronger Coulomb interaction keeps electrons and holes together, giving rise to small dipoles $d_{\text{e-h}} \approx 1$nm comparable with vertical heterostructures [3], cf. the red line in Fig. S1(d). As a result, in the free-standing case, we find for the CT exciton binding energies in the range of 200 meV, cf. the red line in Fig. S1(d) - in agreement with vertical MoS_2-WSe_2 heterostructures [3].

2. ENERGY LANDSCAPE IN HBN-ENCAPSULATED MOSE$_2$-WSE$_2$ HETEROSTRUCTURES

In the main manuscript we have focused on the most relevant bright KK excitons with both electron and hole being localized at the K valley (of different materials in the case of CT excitons). In Fig. S2 we investigate the expected role of momentum-dark excitons. In WSe_2 monolayers, the indirect or momentum-dark KK$'$ and KΛ excitons (also called KQ), formed with a hole in the K valley and an electron in the K$'$ and Λ valley, respectively, are energetically below the bright KK exciton, resulting e.g. in phonon sidebands at cryogenic temperatures [4]. To illustrate this energy alignment, in Fig. S2(a) we plot the minimal energy of dark excitons (yellow and green thin lines for KK$'$ and KΛ, respectively) as a function of space along the lateral heterostructure. These values are compared to the energy of monolayer as well as CT bright excitons (thick lines). All valleys have an additional sub-index i =Mo,W to distinguish between bands in the $MoSe_2$ and WSe_2 layer, respectively. The spatial variations of the minimal energies are obtained microscopically by calculating the energy separation between bright and dark excitons in the monolayer case (by solving the Wannier equation) and by taking a spatial variation of the KK energy, as described in the methods section in the main manuscript. At the WSe_2 side the dark excitons are the energetically lowest states. However, their energy is still larger than the bright exciton on the $MoSe_2$ side, because the energy separation $X_{\text{W}} - X_{\text{Mo}}$ between WSe_2 and $MoSe_2$ monolayer peaks is larger than the bright/dark exciton separation in WSe_2, cf. Fig. S2(a).

In Fig. S2(b,c), we discuss also other possible CT-exciton configurations by considering the single-particle band alignment. Lateral $MoSe_2$-WSe_2 heterostructures are expected to have a type II alignment, with the conduction band minimum located in the $MoSe_2$ layer [2]. In Fig. S2(b) we consider CT excitons formed with the hole in the K valley of WSe_2 and electrons from the K or the Λ valley in $MoSe_2$. Due to the single-particle energy dispersion of $MoSe_2$ [5], the free-CT energy E^0_{CT} of the $\text{K}_\text{W}\text{K}_\text{Mo}$ excitons is clearly smaller than the one of $\text{K}_\text{W}\Lambda_\text{Mo}$ excitons. The energy difference corresponds to $\Delta E^{\text{Mo}}_{\Lambda}$, which is the separation between the Λ and the K valley in $MoSe_2$ ($\Delta E^{\text{Mo}}_{\Lambda} \approx$160 meV [5]). In Fig. 2(c), we consider CT excitons with the hole located in the $MoSe_2$ layer. These have energies much larger than E^0_{CT}, even in presence of a conduction Λ valley energetically slightly below valley K [5, 6]. As a result, the energy of the CT exciton $K_{\text{W}}K_{\text{Mo}}$ considered in our work is much smaller than the energies of all other CT configurations. The situation could be different in lateral heterostructures, where tungsten-based materials have the

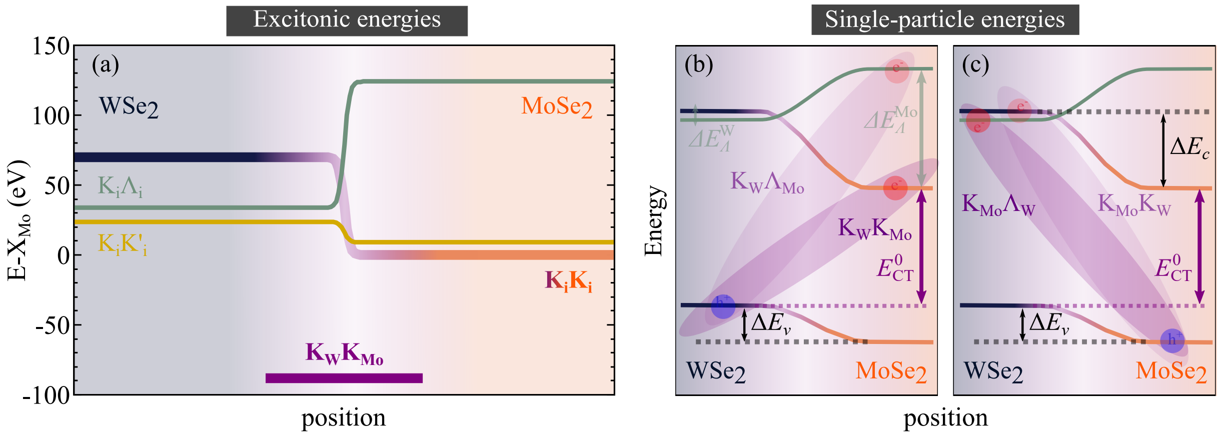

Fig. S2: **Energy landscape in hBN-encapsulated MoSe$_2$-WSe$_2$ heterostructures.** (a) Spatial variation of the minimal excitonic energies for the three key exciton states K_iK_i, $\text{K}_i\text{K}'_i$ and $\text{K}_i\Lambda_i$, with i=Mo,W distinguishing between different valleys in the $MoSe_2$ and WSe_2 layer, respectively. While the momentum-dark excitons ($\text{K}_\text{W}\text{K}'_\text{W}$ and $\text{K}_\text{W}\Lambda_\text{W}$) are the energetically lowest stats in the WSe_2 monolayer, they have a higher energy than the bright exciton $\text{K}_\text{Mo}\text{K}_\text{Mo}$ in the $MoSe_2$ layer due to the larger single particle band-gap in WSe_2. Sketch of the single-particle energy alignment and free-CT energy E^0_{CT} for the configuration with a hole in the K valley of the (b) WSe_2 and (c) $MoSe_2$ layer. Due to the specific alignment, $\text{K}_\text{W}\text{K}_\text{Mo}$ is the CT exciton with the minimal energy.

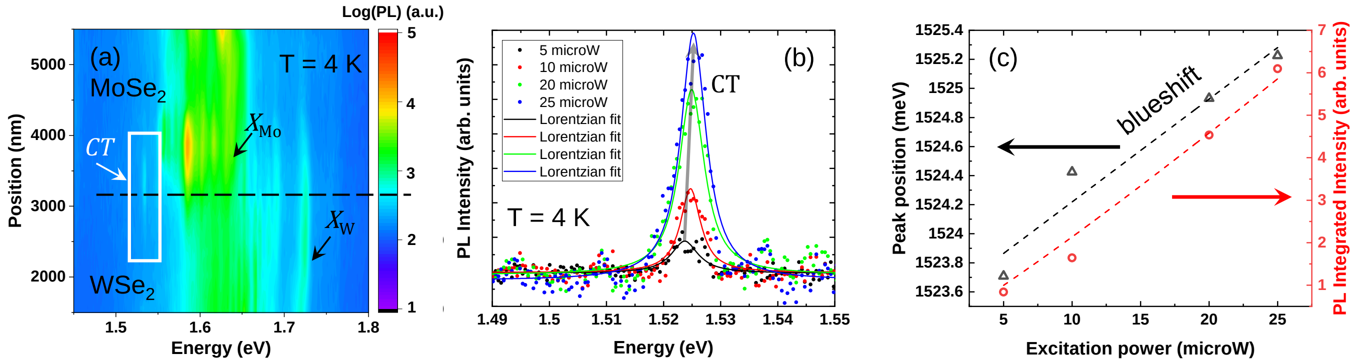

Fig. S3: **Optical measurement of CT excitons** (a) PL linescan across the $MoSe_2/WSe_2$ heterojunction. The position of the heterojunction is indicated by a black dashed line. The step size is 170 nm and the excitation wavelength 633 nm. Neutral excitons of WSe_2, $MoSe_2$ are indicated by black arrows, while the CT exciton is shown by a white box. (b) Power dependence of CT excitons fitted by a Lorentzian. A grey arrow shows a slight blue shift with the increasing excitation power. (c) Peak position (black) and PL integrated intensity (red) as a function of the excitation power. Between 5 μW and 25 μW, we observe a blue-shift of approx. 1.5 meV. A power law of 1.1 suggests linear PL intensity dependence within this range of excitation powers.

minimum conduction band. Given the band alignments between different TMD monolayers [2], this could potentially be the case for WSe_2-WS_2 heterostructure, where we could expect the ground state to be the CT exciton with the electron lying in the K′ or Λ-valley in the WS_2 layer.

3. POWER-DEPENDENT OPTICAL SPECTRA

In Fig. S3 we present optical studies of our samples scanning though the interface (a) or at the interface after increasing the excitation power (b,c). In Fig. S3 we present the PL linescans across $MoSe_2/WSe_2$ heterojunctions at T=4K (Fig. S3). At 1.525eV a peak with a linewidth of approx. 5 meV appears only at the interface (white box in Fig. S3(a)). This raster scanning has been performed on different junctions with respect to the two considered for Fig. 4 in the main manuscript, further confirming the presence of this low energy peak at different interfaces. In this specific junction we also observe a feature slightly below 1.6 eV and roughly 200 nm into the $MoSe_2$ side, probably originating from defects or bubbles close to the interface. The monolayer bright peaks $X_{\rm Mo}$ and $X_{\rm W}$ extend respectively in the $MoSe_2$ and WSe_2 side (black arrows). Energetically below $X_{\rm Mo}$ and $X_{\rm W}$ one can see other features extending over the $MoSe_2$ and WSe_2 side. These are likely induced by trions or by phonon sidebands from dark excitons in WSe_2 [4], cf. Fig. 2.

To better address the origin of the low-energy peak appearing only at the interface, in Figs. 3(b-c) we present power-dependent studies for the same junction as in Fig. S3(a). In Fig. S3(b) we present the spectrally-resolved PL after excitation at the interface and focusing on this low-energy peak. To better appreciate the energetic shape and position of this feature, we also show its associated Lorentzian fits (solid lines in Fig. S3(b)). In Fig. S3(c) we consider more quantitatively this peak in terms of its energy-integrated intensity and energy position. We fit the integrated intensity with a power law function ($y = ax^\beta$) and find a close-to-linear behavior as a function of power (β=1.1, red points in Fig. S3(c)) contrary to the sublinear saturating behavior expected from defect states [7–9]. In the same power range, neutral excitons of $MoSe_2$ and WSe_2 also show a linear dependence.

Similarly, we also fit the emission energy (Fig. S3(c), black points) and find a slight blue-shift as a function of the excitation power. Interestingly, this blue-shift is qualitatively similar to the one found for interlayer excitons [10–13], where it is caused by the dipole-dipole repulsion [14]. The measured blue-shift of the low-energy peak could thus indicate its dipolar origin and further confirm our assignment to CT excitons.

[1] L. Yuan, B. Zheng, Q. Zhao, R. Kempt, T. Brumme, A. B. Kuc, C. Ma, S. Deng, A. Pan, and L. Huang, arXiv preprint arXiv:2111.07887 (2021).
[2] Y. Guo and J. Robertson, Appl. Phys. Lett. **108**, 233104 (2016).
[3] S. Latini, K. T. Winther, T. Olsen, and K. S. Thygesen, Nano Lett. **17**, 938 (2017), ISSN 1530-6984.
[4] S. Brem, A. Ekman, D. Christiansen, F. Katsch, M. Selig, C. Robert, X. Marie, B. Urbaszek, A. Knorr, and E. Malic, Nano Lett. **20**, 2849 (2020), ISSN 1530-6984.
[5] A. Kormányos, G. Burkard, M. Gmitra, J. Fabian, V. Zólyomi, N. D. Drummond, and V. Fal'ko, 2D Mater. **2**, 022001 (2015).
[6] C. Zhang, Y. Chen, A. Johnson, M.-Y. Li, L.-J. Li, P. C. Mende, R. M. Feenstra, and C.-K. Shih, Nano Lett. **15**, 6494 (2015).
[7] T. Schmidt, K. Lischka, and W. Zulehner, Phys. Rev. B **45**, 8989 (1992).
[8] Z. Wu and Z. Ni, Nanophotonics **6**, 1219 (2017).
[9] P. Hernández López, S. Heeg, C. Schattauer, S. Kovalchuk, A. Kumar, D. J. Bock, J. N. Kirchhof, B. Höfer, K. Greben, D. Yagodkin, et al., Nat. Commun. **13**, 7691 (2022).
[10] P. Nagler, G. Plechinger, M. V. Ballottin, A. Mitioglu, S. Meier, N. Paradiso, C. Strunk, A. Chernikov, P. C. M. Christianen, C. Schüller, et al., 2D Mater. **4**, 025112 (2017).
[11] D. Unuchek, A. Ciarrocchi, A. Avsar, Z. Sun, K. Watanabe, T. Taniguchi, and A. Kis, Nat. Nanotechnol. **14**, 1104 (2019).
[12] P. Nagler, F. Mooshammer, J. Kunstmann, M. V. Ballottin, A. Mitioglu, A. Chernikov, A. Chaves, F. Stein, N. Paradiso, S. Meier, et al., Phys. Status Solidi B **256**, 1900308 (2019).
[13] L. Yuan, B. Zheng, J. Kunstmann, T. Brumme, A. B. Kuc, C. Ma, S. Deng, D. Blach, A. Pan, and L. Huang, Nat. Mater. **19**, 617 (2020).
[14] D. Erkensten, S. Brem, R. Perea-Causín, and E. Malic, Phys. Rev. Mater. **6**, 094006 (2022).
